# A Patient-Centered Perspective on Cancer Survivorship

**DOI:** 10.3390/jpm5020091

**Published:** 2015-04-15

**Authors:** Brad Zebrack

**Affiliations:** University of Michigan School of Social Work, 1080 S University, Ann Arbor, MI 48109-1106, USA; E-Mail: zebrack@umich.edu; Tel.: +1-734-615-5940; Fax: +1-734-763-3372

**Keywords:** patient-centered, survivorship, psychosocial, quality of life, qualitative research

## Abstract

Survivorship is a complicated notion because people often confuse a process of survivorship with a mythic identity of being a cancer survivor. This confusion may be a distraction to addressing the real-life struggles and challenges experienced by all people diagnosed with cancer. A more expansive perspective of survivorship, one that attends to patients’ physical, psychological, social, spiritual, and existential challenges throughout a continuum of care, would be more in line with what is known empirically about people’s experiences with cancer. In an effort to gain a patient-centered perspective on cancer, and one that emphasizes multiple dimensions of cancer survivorship, the author reports findings from a non-scientific social media poll (via Facebook and personal emails) in which survivors and colleagues working in the field of cancer survivorship answered the question: What does cancer survivorship mean to you? The comments are enlightening and useful for guiding the development of a patient-centered, and, thus, more comprehensive, approach to caring for people affected by cancer.

## 1. Introduction

Regardless of the type of cancer or the extent of survival, all persons diagnosed with cancer must manage the enduring and complex ways in which cancer transforms the self and everyday life [1].

There was a time when “cancer survivors” were considered the family members, friends and loved ones left behind when someone died of cancer. Today, “cancer survivors” refers to the nearly 14 million individuals in the US who themselves are alive after a cancer diagnosis.

In 2005, the Institute of Medicine emphasized the importance of attending to the lives and quality of life of cancer survivors [2]. The report, “From Cancer Patient to Cancer Survivor: Lost in Transition,” reinforced the notion of survivorship as a distinct phase of cancer treatment characterized by biomedical and psychosocial effects unique to a post-treatment phase. Clinical care and research has been bolstered by this dominant medical perspective that characterizes survivorship as a period of time involving “short- and long-term treatment-related side effects, development of second cancers, as well as psychological and psychosocial perturbations” [3].

In an effort to gain a patient-centered perspective on cancer, and one that emphasizes the psychological, social, and experiential aspects of cancer survivorship, I conducted my own social media poll of friends and colleagues by asking them via Facebook and personal email: What does cancer survivorship mean to you? The comments are enlightening, and I believe useful, for guiding the development of a patient-centered, and thus more comprehensive, approach to caring for people affected by cancer.

Survivorship is a complicated notion because people often confuse the process of survivorship with the mythic identity of being a cancer survivor. Indeed, Dr. Pamela Leung, Professor of Social Work at Hong Kong University, reported: “There is no Chinese translation for the word ‘survivor.’ As more and more people live longer with incurable cancer, or after remission, for substantial periods of time, I think cancer survivorship should not be restricted to those who have their cancer cured” [4]*.*

The National Cancer Institute’s (NCI) Office of Cancer Survivorship states that “an individual is considered a cancer survivor from the time of diagnosis, through the balance of his or her life.” This definition identifies a specific population with its own unique needs and experiences, and also suggests that survivorship challenges and quality of life issues initiate at the time of diagnosis and occur throughout a continuum of care. However, it also confounds an identity—being a cancer survivor—with a medical condition and social phenomenon—an experience of living with cancer. People with cancer have varied and complex feelings of whether or not they feel they are “survivors”.

NCI’s definition of a cancer survivor contrasts with the research portfolio it holds and supports, which focuses solely on a post-treatment phase. This apparent contradiction is evidence that the terms “survivor” and “survivorship” are fraught with ambiguity and require further clarity and theoretically-derived meaning. The confusion may be a distraction to addressing the real-life struggles and challenges experienced by all people diagnosed with cancer.

Lacking cogent definition and theory that distinguishes “survivor” from “survivorship” has led some patients into feeling pigeon-holed into an identity that does not accurately reflect their experience and to which they cannot relate. In spite of a growing movement and number of cancer survivors, many health care providers, as well as patients, do not like the term. Says long-time oncology social work leader, Dr. James Zabora: “I have never really like this term. I believe that survivor has a negative connotation and creates the image of a long-term and difficult struggle. It is as though we had to create another field of practice. I have always favored approaches such as ‘a life with cancer’ or ‘living with cancer’ because I think these terms are more positive and accurate” [4].

As described by self-proclaimed cancer survivor and oncology social worker Richard Dickens, “Survivorship is a paradoxical word eliciting a myriad of meanings, including pride, uncertainty, safety, guilt, good fortune, gratitude, hope, and perhaps the most real and yet most unspoken, not dead. Each of these meanings, like cancer, elicits a myriad of feelings, physically and emotionally” [4]. Mr. Dickens describes his discomfort with the term, due to its ambiguity, as well as many patients’ reticence to acknowledge or afford meaning to the term. “I am always attuned to its resonance and bravado”, he continues, “sometimes humbled by it, never completely owning it…Surviving cancer implies pain, endurance. Maybe that’s what survivorship is: enduring. If one cannot find peace with survivorship, perhaps patients can at least find détente” [4].

Survivorship is NOT simply about being or feeling like a cancer “survivor”. In caring for people with cancer, we must avoid what Heidi Adams, founder of Planet Cancer and Chief Executive Office for Critical Mass, calls the “semantic minefield” of survivorship, which fails to distinguish identity from life experience. A more expansive perspective of survivorship would be more in line with what is known empirically, that early experiences have life-long repercussions. Thus, survivorship care would require expanded attention to patients’ physical, psychological, social, spiritual, and existential challenges throughout a continuum of care. Its goal would be the minimization of long-term debilitation, but also the facilitation of patient coping and adaptation, and perhaps even the promotion of personal transformation and growth.

To talk about cancer survivorship broadens the context for understanding how cancer affects people’s lives, attitudes, and behaviors, and how best to attend to them. Indeed, “Survivorship” can become a framework upon which to build, test, enhance, and reinforce systems of cancer care [1]. It reflects a patient-centered perspective that may be helpful in enhancing people’s experience with cancer, their satisfaction with care, and ultimately their quality of life and survival.

## 2. A Patient-Centered Perspective on Survivorship

A patient-centered perspective on survivorship was first articulated, and probably best epitomized, by Susan Leigh, an oncology nurse, three-time cancer survivor, and founding member of the National Coalition for Cancer Survivorship. She was the first to suggest that cancer survivorship is about “living with, through, and beyond cancer” [5]. This definition acknowledges survivorship as a process of living with cancer that is initiated at diagnosis and continues through phases of treatment and either a transition to off-treatment survival or the end-of-life. It sets us up for understanding and addressing patient and family needs and issues as they occur and re-occur throughout a continuum of care. Furthermore, an existing body of evidence indicates that these risks, experiences, and needs are different for different people depending upon their age at diagnosis, their race, ethnic and cultural background, their socioeconomic status, and the extent and quality of their relationships and social networks.

Social work researcher Dr. Frances Nedjat-Haiem, PhD, offers a patient-centered view of survivorship based on her research involving observations and interviews with underserved and disadvantaged low-income Latino cancer patients in Los Angeles. According to Dr. Nedjat-Haiem, cancer survivorship means [4]:

- Riding the bus to and from medical appointments.
- Waiting in long lines, sometimes all day without being seen by a medical provider.
- Not getting the care you need because you cannot afford to pay for the costs of care.
- Making a choice to see a physician even though you fear being deported.
- Remaining silent when you should speak up because you feel no one really understands you.
- Facing cancer alone because family members are too worried about losing their job if they spend any time away from work.
- Doing whatever it takes to stay alive and be on this earth for your children.

Les Gallo-Silver, long-time oncology social worker and Associate Professor of Health Sciences, described survivorship as “a process of retaining control of one’s life after the diagnosis, making informed decisions about treatment, adapting to changes in daily routines, and developing strategies to manage temporary and perhaps permanent physical changes. Survivorship is not letting cancer define you even as it changes you. Survivorship can be emotionally centering and energize a sense of purpose and an altered direction in one’s life. Medicine may measure survival in weeks, months or years from diagnosis, yet the human spirit measures survival in experiences to be appreciated and intimacies to be cherished” [4].

Oncology social worker Pat Fobair suggested that survivorship is about “[L]earning to look at life in new ways. It is about forgiving friends and family who don’t come through for us, looking at the meaning in life from different perspectives, deciding how you want to proceed, making new plans, opportunities, relationships” [4].

Living is a process. Living with cancer is a process. What happens in early phases of diagnosis and treatment influence later life experiences? Behavioral scientist and quality of life researcher Carolyn Gotay, PhD, suggests that cancer survivorship is “a constantly shifting landscape, not limited to people who are cancer-free or to people who have completed cancer treatment, and involves others beyond the person who has had a cancer diagnosis” [4].

From the point of view of caregivers and loved ones, survivorship is about gratitude and relief, but also fear and disappointment, and how the experience is life-altering for them, as well. Examples of these viewpoints follow:

“It means growing up with a mother! My mom had breast cancer when I was 5 years old. I remember my grandmother taking care of us when she was sick, and her having to wear a wig. She survived and is now 74. I guess you can say my kids have a grandmother, too” [4].

“It has meant more years with a dear friend” [4].

“The fear experienced by me as a caregiver and daughter to my mother with her diagnosis of lung cancer had me wailing and rolling on the floor of my bedroom. Not something I had ever done or have done since” [4].

## 3. Conclusions

Articulating the difference between “survivorship” and “being a survivor” can distinguish a process of living from a state of being or identity. From diagnosis until one’s final breath, individuals with cancer are in a process of survivorship—living with cancer. How different our approach to oncology care, including end-of-life care, might be if we were to integrate survivorship into conventional approaches to clinical care, where both patients and providers meet and negotiate mutual interests and therapeutic goals. Elevating the needs, values, and preferences of patients and their families can only lead to the achievement of greater patient participation in their own care, enhanced mutual decision-making and service delivery, and improved patient outcomes. As for defining who or what is a survivor, perhaps that definition should be left solely to each individual living with cancer.
